# A NuRD Complex from *Xenopus laevis* Eggs Is Essential for DNA Replication during Early Embryogenesis

**DOI:** 10.1016/j.celrep.2018.02.015

**Published:** 2018-02-27

**Authors:** Christo P. Christov, Kevin S. Dingwell, Mark Skehel, Helen S. Wilkes, Julian E. Sale, James C. Smith, Torsten Krude

**Affiliations:** 1 Department of Zoology, University of Cambridge, Downing Street, Cambridge CB2 3EJ, UK; 2 The Francis Crick Institute, Developmental Biology Laboratory, 1 Midland Road, London, NW1 1AT, UK; 3 MRC Laboratory of Molecular Biology, Francis Crick Avenue, Cambridge CB2 0QH, UK

**Keywords:** DNA replication, initiation, *Xenopus laevis*, mid-blastula transition, development, NuRD, chromatin remodeling, histone deacetylase, non-coding RNA, Y RNA

## Abstract

DNA replication in the embryo of *Xenopus laevis* changes dramatically at the mid-blastula transition (MBT), with Y RNA-independent random initiation switching to Y RNA-dependent initiation at specific origins. Here, we identify xNuRD, an MTA2-containing assemblage of the nucleosome remodeling and histone deacetylation complex NuRD, as an essential factor in pre-MBT *Xenopus* embryos that overcomes a functional requirement for Y RNAs during DNA replication. Human NuRD complexes have a different subunit composition than xNuRD and do not support Y RNA-independent initiation of DNA replication. Blocking or immunodepletion of xNuRD inhibits DNA replication initiation in isolated nuclei *in vitro* and causes inhibition of DNA synthesis, developmental delay, and embryonic lethality in early embryos. xNuRD activity declines after the MBT, coinciding with dissociation of the complex and emergence of Y RNA-dependent initiation. Our data thus reveal an essential role for a NuRD complex as a DNA replication factor during early *Xenopus* development.

## Highlights

• xNuRD is essential for DNA replication and *Xenopus* development before the MBT
• xNuRD can functionally substitute for non-coding Y RNAs to initiate DNA replication
• xNuRD complex consists of CHD4, MTA2, HDACm, MBD3, GATAD2, and RBBP4/7 subunits
• Human NuRD complexes cannot support Y RNA-independent DNA replication

## Graphical Abstract

**Figure:**
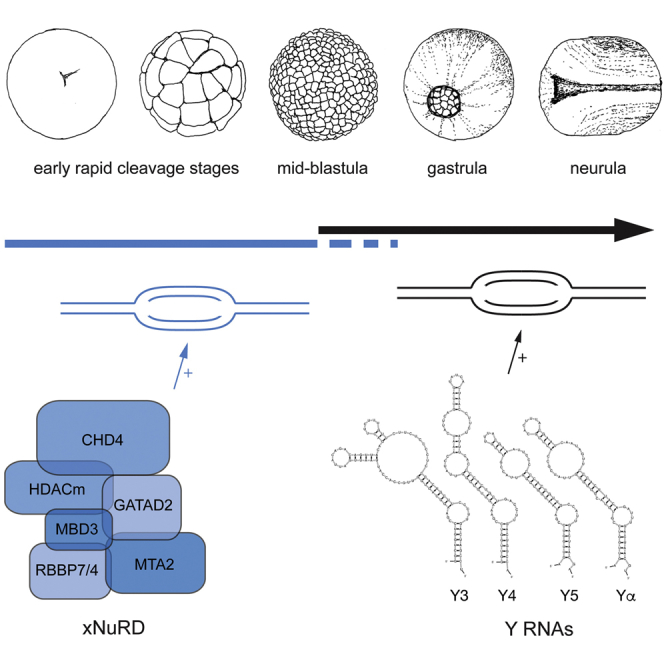

## Teaser

Christov et al. show that the chromatin remodeling complex xNuRD is an essential DNA replication factor in the eggs and early embryos of *Xenopus laevis*. They demonstrate that xNuRD can initiate DNA replication in the absence of non-coding Y RNAs, which only become essential for replication later in development.

## Introduction

Accurate replication of chromosomal DNA is essential for cell proliferation, development, and homeostasis of multicellular organisms. The principles of chromosomal DNA replication are evolutionarily conserved in eukaryotes, but regulation of the initiation of DNA replication changes considerably during early vertebrate development. In *Xenopus laevis*, the mid-blastula transition (MBT) marks a turning point for several features of DNA replication (Hyrien et al., 1995, Lemaitre et al., 1998, Montag et al., 1988, Newport and Kirschner, 1982a, Newport and Kirschner, 1982b). In activated eggs and pre-MBT embryos, cell divisions alternate and even partly overlap with very short S phases, during which tens of thousands of replication forks are established without site specificity and in the absence of transcription. After the MBT, bulk transcription of zygotic genes occurs, and DNA replication initiates at defined replication origins, which often overlap with transcription start sites. S phase and mitosis become separated by G1 and G2 phases, and the duration of S phase extends to several hours.

The molecular mechanisms that underlie this transition of DNA replication control are only now beginning to emerge. In vertebrate somatic cells, initiation of DNA replication depends on small non-coding Y RNAs (Christov et al., 2006, Collart et al., 2011, Kowalski and Krude, 2015, Krude et al., 2009). Intriguingly, even though large amounts of these Y RNAs are maternally deposited, they are not required for DNA replication before the MBT but become essential for DNA replication, cell proliferation, and embryo viability after the MBT (Collart et al., 2011). It is not known how DNA replication can initiate in the absence of Y RNAs during early development.

Here we report the isolation and characterization of a factor from activated *Xenopus laevis* eggs and pre-MBT embryos that allows the initiation of chromosomal DNA replication in the absence of non-coding Y RNAs. To identify this factor, we modified a human cell-free DNA replication initiation system. In this assay, late G1 phase human template nuclei initiate DNA replication *in vitro* in the presence of a cytosolic extract from proliferating HeLa cells, which provides soluble initiation factors, including proteins and Y RNAs (Christov et al., 2006, Krude, 2000, Krude et al., 1997). Degradation of endogenous Y RNAs in the cytosolic extract inhibits the initiation of DNA replication in this system (Christov et al., 2006, Krude et al., 2009). In contrast, although *Xenopus laevis* egg extracts also cause initiation of DNA replication in human late G1 phase nuclei *in vitro*, degradation of endogenous *Xenopus* Y RNAs does not inhibit initiation activity (Collart et al., 2011), suggesting that there is a Y RNA-independent initiation activity in *Xenopus* egg extracts.

Chromatin is rendered dynamic by the activities of histone-modifying enzymes (Kouzarides, 2007) and by ATP-dependent chromatin remodeling factors that affect the composition and position of nucleosomes along chromosomal DNA (Clapier and Cairns, 2009). The chromatin remodeling factor NuRD (nucleosome remodeling and deacetylase) combines two important chromatin-modifying activities (Allen et al., 2013, Ho and Crabtree, 2010, Laugesen and Helin, 2014, Torchy et al., 2015). First, ATP-dependent nucleosome remodeling is catalyzed by the ATPase/helicase activities of the large CHD3/4 subunits (chromodomain helicase DNA-binding 3/4). In addition, NuRD has lysine deacetylation activity, which is provided by the HDAC1/2 subunits (histone deacetylase 1/2). Four additional subunits promote interactions with DNA, histones, and transcriptional regulators: MTA1/2/3 (metastasis-associated 1/2/3) interact with transcriptional regulators and histones; MBD2/3 (methyl-CpG binding domain 2/3) interact with methylated DNA; and GATAD2A/B (GATA zinc-finger domain containing 2A/B, also known as p66α/β) and RBBP7/4 (retinoblastoma binding proteins 7/4, also known as (RbA)p46/p48) interact with histones.

Importantly, each canonical NuRD subunit has two or three homologs, some of which are present only in mutually exclusive NuRD complexes. This allows the formation of a large number of different complexes with different biological functions, which include the regulation of transcription, maintenance of genome stability, embryonic development, and cancer (Allen et al., 2013, Ho and Crabtree, 2010, Laugesen and Helin, 2014, Torchy et al., 2015). However, no NuRD complex has yet been implicated in the regulation of DNA replication.

Here we identify a maternally deposited NuRD complex as a DNA replication factor in *Xenopus* eggs and early embryos that can overcome the requirement for Y RNAs to initiate chromosomal DNA replication.

## Results

### A Y RNA-Independent Initiation Activity from *Xenopus* Egg Extracts

**Figure 1:**
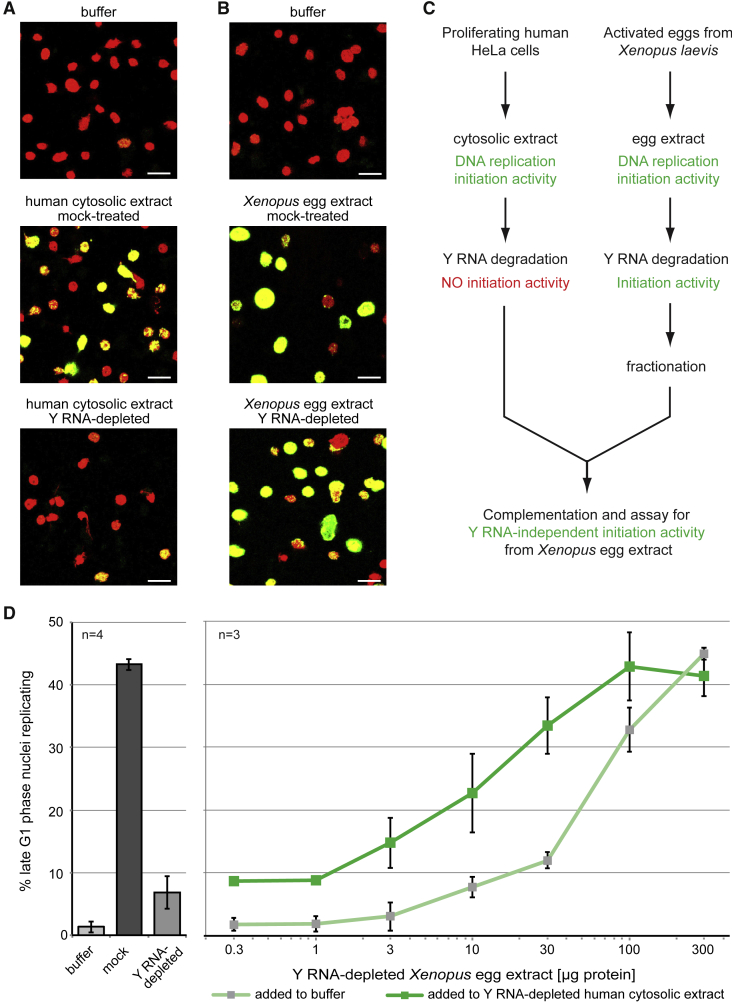
Y RNA-Independent Initiation Activity in *Xenopus* Egg Extracts (A) Y RNA-dependent initiation of DNA replication in a human cell-free system. Human late G1 phase template nuclei were incubated in either buffer (top) or mock-treated or Y RNA-depleted cytosolic extracts. (B) Y RNA-independent initiation of DNA replication in *Xenopus* egg extracts. Human late G1 phase template nuclei were incubated in either buffer (top) or mock-treated or Y RNA-depleted *Xenopus* egg extracts. Representative immunofluorescence micrographs are shown with merged channels for total DNA (propidium iodide, red) or sites of DNA replication (digoxigenin-deoxyuridine triphosphate [dUTP] incorporation, green). (C) Experimental design for the detection and isolation of the initiation activity from *Xenopus* egg extracts by cross-species complementation assays. (D) Cross-species complementation assays. Left: template nuclei were incubated as in (A) in either buffer or mock-treated cytosolic or Y RNA-depleted human cytosolic extracts. Right: increasing amounts of Y RNA-depleted *Xenopus* egg extracts were added to replication initiation reactions in the presence of either buffer (light green) or Y RNA-depleted human cytosolic extract (dark green). Percentages of replicating template nuclei for each reaction are shown as mean values ± SD of n independent experiments. Scale bars, 10 μm.

The initiation step of chromosomal DNA replication can be reconstituted in a human cell-free system in which late G1 phase template nuclei are incubated in a cytosolic extract from proliferating cells (Krude, 2000). Degradation of all four human Y RNAs from such an extract causes a 5- to 10-fold reduction in the number of replicating nuclei (Figures 1A and 1D), consistent with an essential role for human Y RNAs as initiation factors in this system (Christov et al., 2006). This Y-RNA-depleted system forms the basis of a screen for activities that might replace Y RNAs.

Human late G1 phase template nuclei also initiate DNA replication when incubated in an extract from activated *Xenopus laevis* eggs (Figure 1B). Degradation of the four endogenous Y RNAs from the egg extract, however, does not lead to inhibition of DNA replication (Figure 1B), consistent with the observation that Y RNAs are not required for DNA replication in activated eggs and early embryos (Collart et al., 2011). Significantly, addition of Y RNA-depleted *Xenopus* egg extract to Y RNA-depleted human cytosolic extract increases the percentages of replicating G1 phase template nuclei in a dose-dependent manner above that observed using egg extract alone (Figures 1C and 1D). This indicates that Y RNA-depleted *Xenopus* egg extract contains an activity that can initiate chromosomal DNA replication in human cell nuclei in the absence of Y RNAs. Treatments of the egg extract with heat, proteinase K, and/or phenol inactivated the activity but treatment with RNase A did not (data not shown), suggesting that it is protein-associated. We set out to isolate this initiation activity by systematic fractionation of the egg extract.

### Isolation of the Y RNA-Independent Initiation Activity

**Figure 2:**
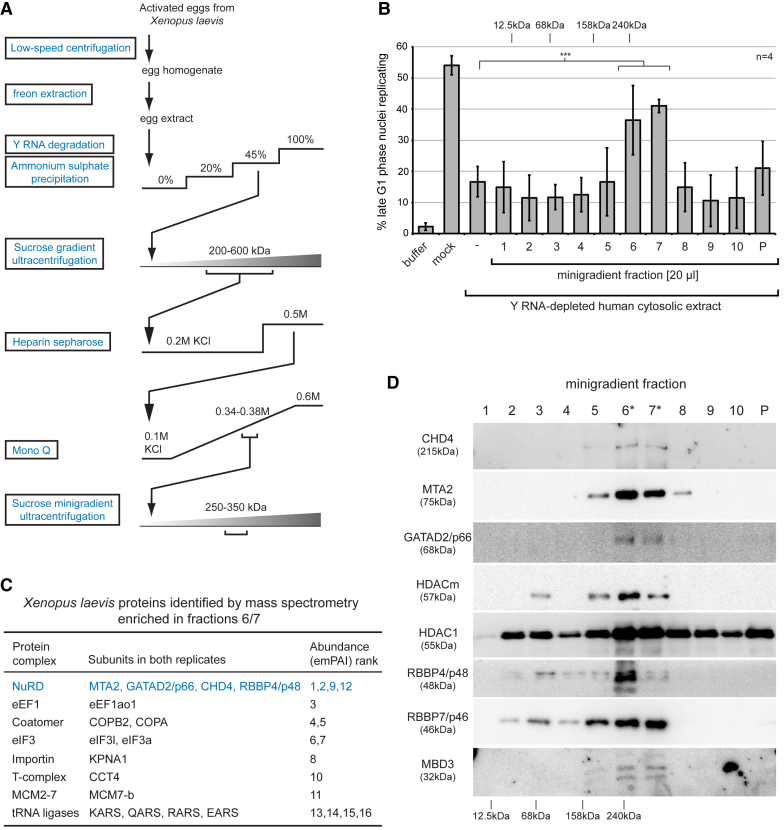
Isolation and Identification of the Y RNA-Independent Initiation Factor from *Xenopus* Egg Extracts (A) Schematic representation of the fractionation scheme. (B) Activity profile of the sucrose minigradient fractions. Template nuclei were incubated in Y RNA-depleted human cytosolic extract supplemented with the indicated gradient fractions. Percentages of replicating nuclei are shown as mean values ± SD of n = 4 independent fractionation experiments. Brackets indicate results of t tests (unpaired, two-tailed with unequal variance) of experimental samples (fractions 6 and 7) against the control (^∗∗∗^p ≤ 0.001). Tests of all other fractions against the control were not significant. Sedimentation of calibrator protein complexes (cytochrome *c*, 12.5 kDa; BSA, 68 kDa; aldolase, 158 kDa; catalase, 240 kDa) are indicated at the top. (C) Proteins in active fractions as identified by mass spectrometry. Proteins identified in both of two independent experimental replicates that were enriched in the active gradient fractions 6/7 compared with inactive fraction 5 are ranked according to their abundance (determined as emPAI scores). (D) NuRD subunit sedimentation profiles. Fractions of the sucrose minigradient were analyzed by western blotting with antibodies specific for the indicated NuRD subunits. See also Figure S1 and Table S1.

Isolation of the initiation activity from *Xenopus* egg extracts was achieved by means of seven fractionation steps (Figure 2A). Activated eggs were first crushed by centrifugation, and yolk and cellular debris were extracted with Freon. Endogenous Y RNAs were then degraded by means of endogenous RNase H activity by addition of specific antisense DNA oligonucleotides (Collart et al., 2011). Initiation activity precipitated between 20%–45% ammonium sulfate and was isolated as a single broad peak of 200–600 kDa by ultracentrifugation through a preparative sucrose gradient, thereby removing small proteins and larger ribosomal and spliceosomal complexes. Activity was then partially purified over heparin Sepharose and Mono Q anion exchange columns before ultracentrifugation through a sucrose minigradient, where it sedimented as a narrow peak with an apparent molecular mass of about 250–350 kDa (Figure 2B, fractions 6 and 7). Protein analysis of the gradient fractions indicated that a set of 10–15 major polypeptides co-sedimented with the peak of activity (Figure S1), suggesting that it is a multisubunit protein complex.

To identify proteins present in the active fractions, we subjected them to quantitative mass spectrometry, using a neighboring inactive fraction as a negative control. In two independent purification runs, 183 and 74 unique *Xenopus* proteins were identified in the active fraction from the Uniprot_080713 database (Table S1). After assessing relative abundance by calculating exponentially modified protein abundance index (emPAI) scores, these proteins were ranked on relative enrichment in the active over the inactive fractions and overall abundance. Of the 17 proteins that were enriched in both experimental replicates, we identified four subunits of the nucleosome remodeling and deacetylation complex NuRD: MTA2, GATAD2/p66, CHD4, and RBBP4/p48 (Figure 2C). Additional NuRD subunits—MBD3, RBBP7/p46, MTA1, and the maternal histone deacetylase HDACm (Ryan et al., 1999), also known as probable histone deacetylase 1-A (HDAC1-a)—were also enriched in the active fractions but only detected in one of the two purification runs (Table S1). Individual subunits of several other large multisubunit protein complexes were also identified (Figure 2C; Table S1). The lack of the pre-RC components ORC1-6, Cdt1, Cdc6, and most of MCM2-7 in the active fractions indicates that the isolated initiation activity is required for a post-licensing step of DNA replication initiation, consistent with the presence of pre-replicative complexes (preRCs) in the template nuclei (Krude, 2000, Kubota et al., 2014). Western blotting confirmed the enrichment of all NuRD subunits in the active fractions of the minigradient (Figure 2D). We conclude that a multisubunit NuRD complex co-purifies with the DNA replication initiation activity isolated from activated *Xenopus laevis* eggs.

### The *Xenopus* NuRD Complex Has DNA Replication Initiation Activity

**Figure 3:**
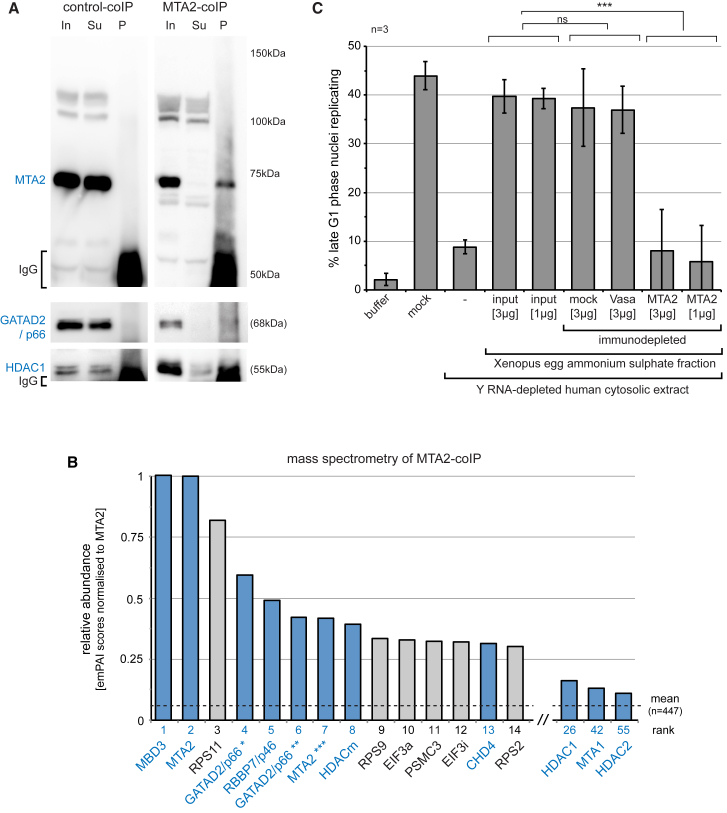
The *Xenopus* NuRD Complex Has DNA Replication Initiation Activity NuRD was immunoprecipitated with MTA2-specific antibodies from the 20%–45% ammonium sulfate fraction of the activated *Xenopus laevis* egg extract. (A) Co-immunoprecipitation of GATAD2/p66 and HDAC1 subunits with MTA2, as confirmed by western blotting. Input (In), supernatant (Su), and washed pellet (P) of the control and MTA2 coIPs are shown, 10% of the total was loaded per lane for each. (B) Mass spectrometry analysis of co-immunoprecipitated material. *Xenopus* proteins (n = 447) uniquely identified only in the immunoprecipitate and not in the control IP were ranked according to their quantitative emPAI values after normalization to emPAI (MTA2) = 1. The top 14 most abundant proteins are plotted together with additional NuRD subunits. NuRD subunits are shown in blue. The mean normalized emPAI value of all proteins is indicated by a dashed line. Separate and additional isoforms were identified in the Uniprot database for GATAD2/p66 (^∗^LOC398154; ^∗∗^LOC100158394, isoform X2) and MTA2 (^∗∗∗^MGC83056), respectively. (C) Immunodepletion. xNuRD was co-immunoprecipitated with MTA2-specific antibodies as indicated above. Control immunoprecipitations were performed with either empty beads (mock) or with antibodies specific for *Drosophila* Vasa protein. The indicated protein amounts of immunodepleted supernatants were added to Y RNA-depleted DNA replication initiation reactions. Mean values ± SD of percentages of replicating template nuclei are plotted from n = 3 independent experiments. Brackets indicate results of t tests (unpaired, two-tailed with unequal variance) of experimental and control samples against the untreated input samples (ns, not significant; ^∗∗∗^p ≤ 0.001). See also Table S2.

To assess independently whether this NuRD complex contains the DNA replication initiation activity, we performed immunoprecipitation and functional immunodepletion analyses (Figure 3).

Antibodies specific for MTA2 effectively precipitated MTA2 from the 20%–45% ammonium sulfate fraction of the activated *Xenopus laevis* egg extract whereas unspecific control antibodies did not (Figure 3A). Western blotting confirmed that GATAD2/p66 and HDAC1 co-precipitated with MTA2 under these conditions (Figure 3A). Quantitative mass spectrometry analysis showed that all six subunits of the NuRD complex that were originally isolated by fractionation of the egg extract co-precipitated with MTA2 (Figure 3B; Table S2). These data suggest that a NuRD complex assembled around MTA2 is present in *Xenopus* egg extracts, which we term xNuRD (comprising CHD4, MTA2, GATAD2/p66, HDACm, RBBP7/p46, and MBD3 subunits).

We used immunodepletion to test whether xNuRD contributes to the DNA replication initiation activity with which it co-fractionates. Immunodepletion using MTA2 beads completely abolished initiation activity, whereas control depletions had no effect (Figure 3C). We conclude that xNuRD constitutes an activity that can initiate chromosomal DNA replication in human G1 phase nuclei in the absence of Y RNAs.

### Human NuRD Complexes Are Structurally and Functionally Distinct from xNuRD

**Figure 4:**
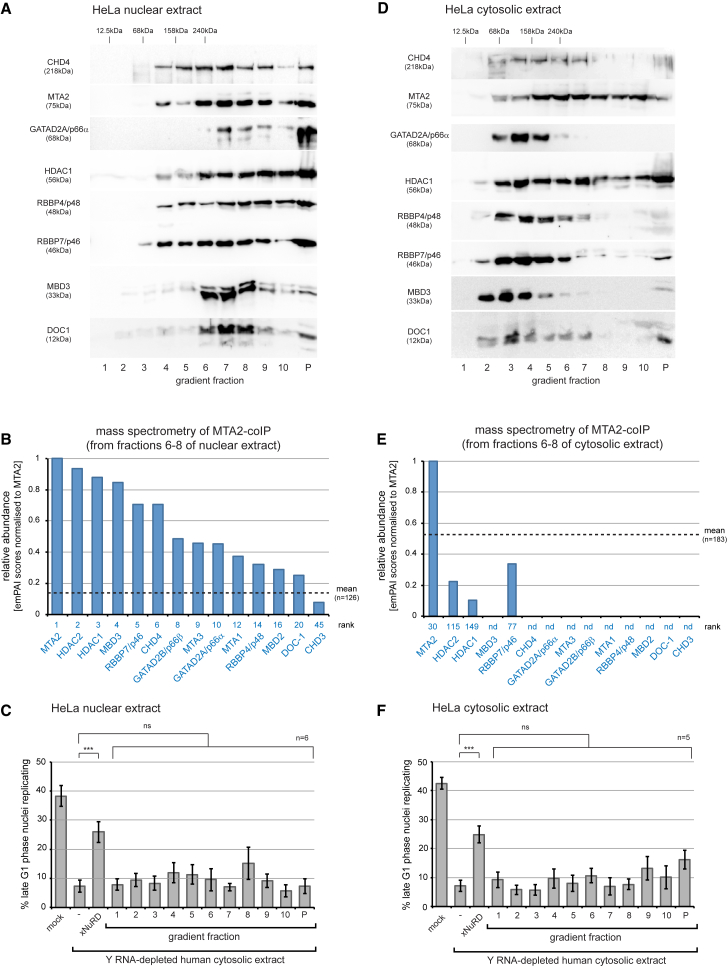
Human NuRD Complexes Are Structurally and Functionally Distinct from xNuRD (A–F) Proliferating human HeLa cells were fractionated into nuclear (A–C) and cytosolic extracts (D–F), and each extract was partially sub-fractionated by precipitation with 20%–45% ammonium sulfate and ultracentrifugation through preparative sucrose gradients. (A and D) Western blot analyses of human NuRD subunits. Fractions of the preparative sucrose gradients of nuclear (A) and cytosolic extracts (D) were analyzed by western blotting with antibodies specific for the indicated human NuRD subunits. (B and E) Mass spectrometry analysis of the MTA2 co-immunoprecipitations. Fractions 6–8 of the preparative sucrose gradients of nuclear (B) and cytosolic extracts (E) were pooled, and human NuRD was immunoprecipitated with anti-MTA2 antibodies. Human proteins identified in the immunoprecipitates (n = 126 and n = 183 for nuclear and cytosolic extracts, respectively) were ranked according to their quantitative emPAI values after normalization to emPAI (MTA2) = 1, and all human NuRD subunits detected are plotted with their rank. The mean normalized emPAI values of all proteins are indicated by dashed lines. (C and F) Activity profiles of the sucrose fractions. Template nuclei were incubated in Y RNA-depleted human cytosolic extract supplemented with the indicated gradient fractions of the nuclear (C) and cytosolic extracts (F). Mean values ± SD of percentages of replicating template nuclei are plotted of n independent experiments. Brackets indicate results of t tests (unpaired, two-tailed with unequal variance) of the positive control (xNuRD) and experimental samples against the Y RNA-depleted background (^∗∗∗^p ≤ 0.001). See also Figures S2 and S3 and Table S3.

NuRD is an evolutionarily conserved protein complex, and several isoforms of each of its subunits are present in human cells (Allen et al., 2013, Torchy et al., 2015). The human Y RNA-depleted cell-free system is thus likely to contain human NuRD (hNuRD). We therefore tested whether hNuRD can substitute for xNuRD in the absence of Y RNAs (Figure 4).

Proliferating HeLa cells were separated into nuclear and cytosolic extracts that were then subjected to Y RNA degradation and further fractionation. Individual hNuRD subunits were present over a range of molecular masses, including a complex with an apparent mass of about 300 kDa in nuclear extracts (Figure 4A, fractions 6–8). A similar complex was observed in nuclear extracts from human embryonic stem cells (hESCs), although several subunits were present in lower-molecular-weight complexes (Figure S2A). Quantitative mass spectrometry showed that several isoforms of each hNuRD subunit effectively co-immunoprecipitated with MTA2 from HeLa nuclear extracts (Figure 4B; Table S3). hNuRD contained MTA1 and MTA3 in addition to MTA2, HDAC2 in addition to HDAC1, isoforms A and B of GATAD2, MBD2 in addition to MBD3, and also the small accessory subunit DOC1/CDK2AP1 (deleted in oral cancer 1/Cdk2-associated protein 1) (Figure 4B; Table S3). This indicates that immunoprecipitated hNuRD complexes show greater subunit diversity than the corresponding xNuRD complex. Importantly, none of the gradient fractions containing nuclear hNuRD subunits from either HeLa cells or hESCs were able to substitute functionally for xNuRD or Y RNAs in the initiation of DNA replication *in vitro* (Figures 4C and S2C), even at up to 4-fold increased amounts compared with xNuRD (data not shown).

Human NuRD was also present in cytosolic extracts of proliferating HeLa cells and hESCs, but with the exception of MTA2, HDAC1, and RBBP4/7, all subunits sedimented through sucrose gradients as predominantly lower-molecular-weight entities (Figures 4D and S2B). Only HDAC2, HDAC1, and RBBP7 co-immunoprecipitated with MTA2 in the high-molecular-weight fractions (Figure 4E; Table S3), confirming that hNuRD is not present as a canonical protein complex in the soluble HeLa cell extract. Importantly, none of the gradient fractions containing cytosolic hNuRD subunits was able to substitute functionally for xNuRD or Y RNAs in the initiation of DNA replication *in vitro* (Figures 4F and S2D).

The lack of an initiation activity associated with hNuRD could be explained by the presence of an inhibitor. To address this possibility, we added hNuRD (fractions 3/4 and 7/8 of cytosolic and nuclear HeLa cell extracts, respectively) to DNA replication reactions driven by xNuRD. No reduction of the initiation frequency was observed (Figure S3), strongly suggesting that hNuRD is not associated with an inhibitor.

We conclude that human NuRD complexes present in HeLa and hESCs are structurally and functionally different from *Xenopus* xNuRD because they exist in different subunit compositions and they cannot substitute for Y RNA activity in the initiation of chromosomal DNA replication in human cell nuclei.

### Inhibition of xNuRD Inhibits Y RNA-Independent DNA Replication

**Figure 5:**
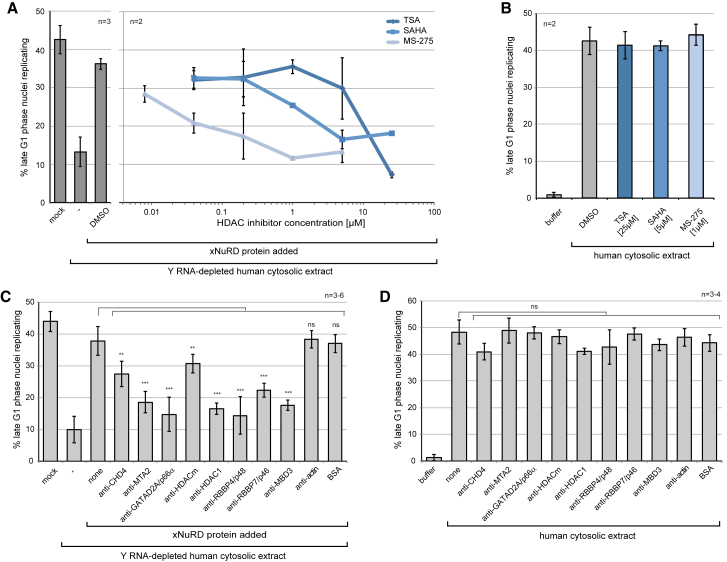
Inhibition of xNuRD Leads to an Inhibition of Y RNA-Independent DNA Replication Shown is inhibition of DNA replication using HDAC inhibitors and NuRD-specific antibodies. (A) Template nuclei were incubated in Y RNA-depleted human cytosolic extract supplemented with partially purified xNuRD and the indicated concentrations of the HDAC inhibitors trichostatin A (TSA), SAHA, and MS-275. (B) Template nuclei were incubated with human cytosolic extract containing human NuRD and Y RNAs in the presence of the indicated inhibitors. (C) Template nuclei were incubated in Y RNA-depleted human cytosolic extract supplemented with partially purified xNuRD and the indicated NuRD-specific antibodies. (D) Template nuclei were incubated with human cytosolic extract containing human NuRD and Y RNAs in the presence of the indicated NuRD-specific antibodies. Mean values ± SD of the percentages of replicating template nuclei are plotted from n independent experiments. Results of t tests (unpaired, two-tailed with unequal variance) of treated experimental samples against the no addition controls are indicated (^∗∗^p ≤ 0.01; ^∗∗∗^p ≤ 0.001).

The class I histone deacetylases HDACm and HDAC1 are present in active xNuRD, and it is possible that they are key subunits for the activity of xNuRD in the initiation of DNA replication. We therefore analyzed their functional requirement by inhibition experiments (Figure 5). Addition of the HDAC inhibitors trichostatin A (TSA), suberoylanilide hydroxamic acid (SAHA), and MS-275 caused a dose-dependent inhibition of the xNuRD-dependent initiation of DNA replication in human cell nuclei *in vitro* (Figure 5A). The increased specificity of the compounds, from HDAC-generic (TSA) via intermediate (SAHA) to HDAC1-specific (MS-275) (Choudhary et al., 2009), correlated with a decreased half maximal inhibitory concentration (IC_50_) over several orders of magnitude (Figure 5A). We conclude that HDACm and/or HDAC1 are essential for xNuRD activity in this DNA replication initiation assay. In contrast, high concentrations of these three HDAC inhibitors had no effect on Y RNA-dependent initiation in the absence of xNuRD (Figure 5B).

Binding of antibodies to proteins can inhibit their function by steric hindrance. We therefore used a panel of antibodies capable of detecting *Xenopus* and human NuRD subunits for functional inhibition studies (Figures 5C and 5D). As a control, addition of either anti-actin antibodies or BSA had no effect on the xNuRD-dependent initiation of DNA replication *in vitro* (Figure 5C). Importantly, antibodies against MTA2, GATAD2/p66, HDAC1, RBBP4/7, and MBD3 all strongly inhibited DNA replication, whereas anti-CHD4 and anti-HDACm antibodies had only a moderate but nevertheless significant inhibitory effect. None of these antibodies inhibited DNA replication in the Y RNA-dependent system in the absence of xNuRD (Figure 5D).

We conclude that xNuRD has a specialist function in Y RNA-independent DNA replication, an activity that is not shared by human NuRD complexes.

### Initiation Activity of xNuRD Declines after the MBT

**Figure 6:**
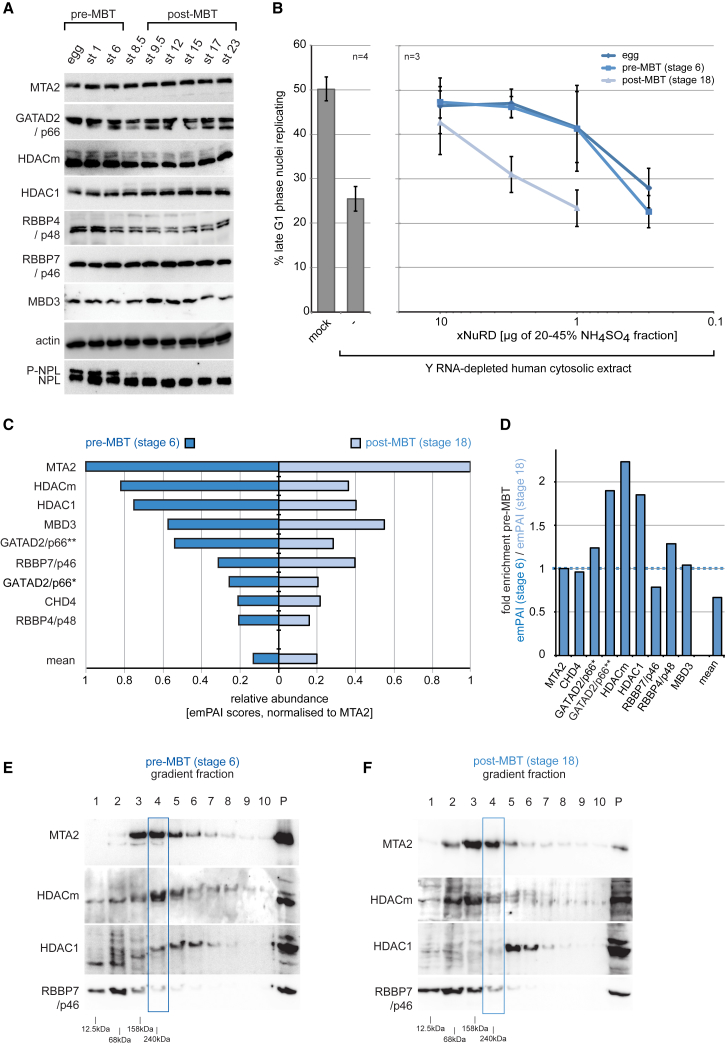
The Replication Initiation Activity and Complex Formation of xNuRD Declines after MBT (A) Expression of xNuRD during development of *Xenopus laevis*. Extracts of staged embryos were analyzed by western blotting. Pre-MBT and post-MBT stages are indicated. Dephosphorylation of hyperphosphorylated nucleoplasmin (P-NPL) is a control for MBT (stage 8.5). (B) DNA replication initiation activity of xNuRD declines after MBT. Template nuclei were incubated in Y RNA-depleted human cytosolic extract supplemented with partially purified xNuRD from activated eggs, stage 6 pre-MBT embryos, and stage 18 post-MBT embryos, as indicated. Mean values ± SD of the percentages of replicating template nuclei are plotted from n independent experiments. (C) Mass spectrometry analyses of xNuRD complexes. MTA2 was immunoprecipitated from pre-MBT (stage 6) and post-MBT (stage 18) embryo extracts. *Xenopus* proteins identified in the immunoprecipitates were ranked according to their quantitative emPAI values in the pre-MBT IP after normalization to emPAI (MTA2) = 1. All xNuRD-specific subunits detected are plotted (left, dark blue bars for pre-MBT; right, light blue bars for post-MBT). The mean normalized emPAI values for all detected proteins are indicated. Separate isoforms were identified in the Uniprot database for GATAD2/p66 (^∗^LOC398154; ^∗∗^LOC100158394, isoform X2). (D) Relative enrichment of xNuRD subunits before MBT in immunoprecipitated complexes. Normalized emPAI values were taken from MTA2 co-immunoprecipitations of stage 6 pre-MBT and stage 18 post-MBT extracts, and their ratios are plotted for the indicated xNuRD subunits and the overall mean. (E and F) xNuRD complex formation in (E) pre-MBT and (F) post-MBT embryo extracts. xNuRD was partially sub-fractionated by precipitation with 20%–45% ammonium sulfate and ultracentrifugation through preparative sucrose gradients. MTA2, HDAC, and RBBP7 subunits were analyzed by western blot analysis of the preparative sucrose gradient fractions. A blue box shows the reference for the sedimentation of active xNuRD from egg extracts. Positions of sedimentation markers are indicated. See also Figure S4 and Table S4.

DNA replication during the early development of *Xenopus laevis* becomes dependent on Y RNA function only after the MBT (Collart et al., 2011). We therefore asked whether xNuRD might be downregulated at this developmental stage (Figure 6).

The dephosphorylation of nucleoplasmin at the MBT (Bürglin et al., 1987, Leno et al., 1996) was used as a marker of developmental stage (Figure 6A). Strikingly, all xNuRD subunits were present throughout early development, and none showed any significant changes in mobility or abundance around the MBT (Figure 6A).

To investigate whether the specific DNA replication initiation activity of xNuRD changes during early development, we partially purified xNuRD from stage 6 pre-MBT and stage 18 post-MBT embryos and compared their specific activities with similarly prepared material from activated eggs. xNuRD preparations from activated eggs and from pre-MBT embryos were similarly active to initiate DNA replication in human G1 phase nuclei, whereas xNuRD from post-MBT embryos was about 10-fold less active (Figure 6B). Because the overall abundance of individual NuRD subunits did not change at MBT (Figure 6A), we investigated, by co-immunoprecipitation (coIP) and mass spectrometry, whether this decline in activity post-MBT corresponds to decreased complex formation (Figure 6C; Table S4). All xNuRD subunits co-precipitated with MTA2 in pre-MBT stage 6 and post-MBT stage 18 embryo extracts, but after normalization to MTA2, the relative abundances of HDACm, HDAC1, and one isoform of GATAD2/p66 were reduced after the MBT (Figures 6C and 6D).

To corroborate these data, we investigated xNuRD complexes by ultracentrifugation through sucrose gradients and western blotting (Figures 6E and 6F). In stage 6 embryo extracts, the majority of MTA2 and HDACm peaked at the position of the entire xNuRD complex (Figure 6E, boxed), which corresponds to xNuRD similarly isolated from activated egg extracts (Figure S4). In contrast, these individual subunit peaks clearly separated from each other in stage 18 embryo extracts (Figure 6F), indicating that xNuRD complex formation is decreased after the MBT.

We conclude that the DNA replication initiation activity of xNuRD is downregulated after the MBT, concurrent with complex dissociation and the emergence of a requirement for non-coding Y RNAs (Collart et al., 2011).

### xNuRD Is Required for Embryo Development before MBT

**Figure 7:**
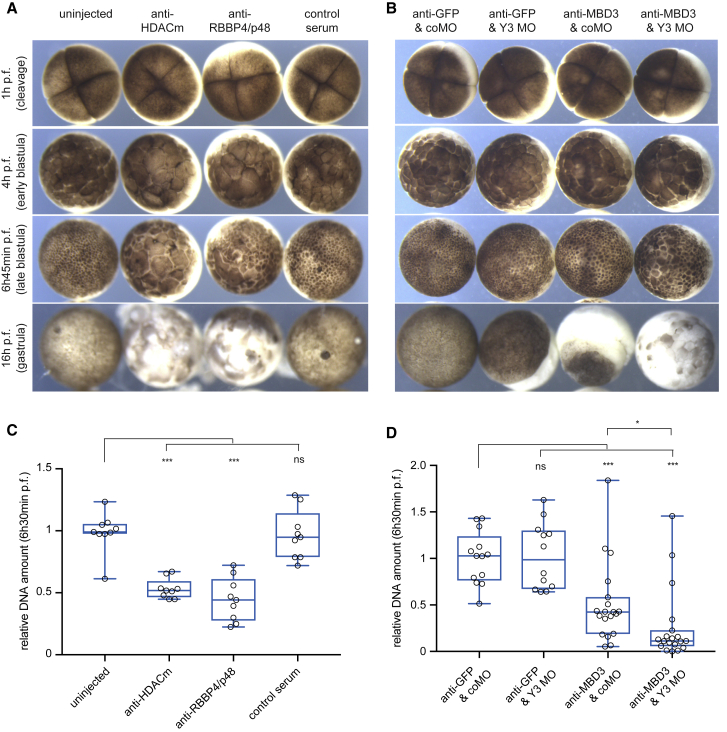
xNuRD Is Required for Development and DNA Replication before the MBT *In Vivo* (A and B) Phenotypes of developing *Xenopus* embryos after microinjection of NuRD-specific antibodies and Y3 RNA-specific antisense morpholino oligonucleotides (MOs). The indicated antibodies (panel A, 65ng serum/embryo; panel B, 5ng IgG/embryo) and MOs (control coMO or Y3 MO, 40 ng/embryo) were injected into the animal pole at the 1-cell stage, and representative embryos were photographed at the indicated times post fertilization (p.f.). See Movies S1 and S2 for the full time course of these experiments. (C and D) DNA replication before the MBT depends on xNuRD. Total embryonic DNA of experimental repeats shown in (A) and (B) was quantified relative to rRNA (C) and (D), respectively. Individual datasets were normalized to the mean of the control distributions and plotted as box and whisker plots superimposed with individual data points. Antibodies available to other xNuRD subunits were ineffective in this assay, regardless of whether Y RNA was co-depleted (Figure S6). Results of t tests (unpaired, two-tailed with unequal variance) of treated experimental samples against the controls are indicated (^∗^p = 0.07; ^∗∗∗^p ≤ 0.001). See also Figures S5 and S6 and Movies S1 and S2.

Finally, we investigated the requirement of xNuRD for DNA replication *in vivo* by inhibiting the xNuRD complex in fertilized *Xenopus laevis* eggs and following the effects during development (Figure 7; Movies S1 and S2). To block xNuRD, we injected antibodies rather than morpholino oligonucleotides (MOs) into fertilized eggs because the long half-life of maternally deposited NuRD protein subunits during early development (Peshkin et al., 2015) would make mRNA targeting via MOs ineffective.

Injection of HDACm- and RBBP4-specific antisera inhibited development before the MBT, resulting in fewer, larger, and irregularly shaped cells at a time when control embryos had reached the early blastula stage (Figure 7A; Movie S1). These embryos arrested development and died at a time when control embryos underwent gastrulation. Injection of MBD3-specific immunoglobulin Gs (IgGs) gave a similar phenotype involving developmental delay and early embryonic death (Figure 7B; Movie S2).

In contrast, functional depletion of non-coding Y3 RNA by injection of antisense MOs (Y3 MOs) produced no phenotype until the MBT but then resulted in developmental arrest and embryonic death during gastrulation (Figure 7B; Movie S2), as described previously (Collart et al., 2011). Simultaneous injection of Y3 MO and MBD3-specific IgGs resulted in developmental delay before the MBT, as seen with inhibition of MBD alone, but the severity of the phenotype was increased and the onset of embryonic death accelerated (Figure 7B; Movie S2). In contrast, control embryos continued to develop normally (Figure S5).

Finally, we asked whether the developmental delay and embryonic death caused by xNuRD inhibition is due to an inhibition of DNA replication. Injection of HDACm-, RBBP4-, and MBD3-specific antibodies resulted in a severe inhibition of DNA synthesis before the MBT (Figures 7C and 7D). As a control, functional depletion of Y3 RNA by injection of Y3 MO had no effect on DNA replication before the MBT (Figure 7D), as described previously (Collart et al., 2011). However, co-injection of Y3 MO with MBD3-specific antibodies moderately increased the inhibition of DNA synthesis obtained with MBD3-specific antibodies alone (Figure 7D), suggesting that xNuRD and Y RNAs may operate side by side around the MBT.

Taken together, we conclude that the xNuRD complex is essential for DNA replication and embryo development before the MBT and that xNuRD acts prior to the requirement for non-coding Y RNAs in early embryos.

## Discussion

In this study, we have isolated an isoform of the nucleosome remodeling and histone deacetylation complex NuRD as a DNA replication initiation factor from activated eggs and early pre-MBT embryos of *Xenopus laevis* that can overcome a functional requirement for non-coding Y RNAs. NuRD has been implicated as a repressor of transcription in various systems, and here we demonstrate an additional and essential function for DNA replication in early *Xenopus* embryos.

### Combinatorial Assembly of Functional NuRD Complexes

NuRD comprises a class of evolutionarily conserved eukaryotic protein complexes. They form by combinatorial assembly from six canonical subunits that are each encoded by gene families, including CHD3/4, MTA1/2/3, HDAC1/2, RBBP4/7, and MBD2/3 (Allen et al., 2013, Torchy et al., 2015). The presence of only one particular gene family product for each subunit is often characteristic of a particular NuRD complex. The xNuRD complex we have isolated is no exception because we found CHD4 (and not CHD3), MTA2 (and not MTA1 or 3), HDACm/HDAC 1 (and not HDAC 2), and MBD3 (and not MBD2) as constituents. In addition, we found two isoforms of GATAD2 and both RBBP4/7, suggesting that these two subunits may be present in a non-exclusive manner.

The stoichiometry of NuRD complexes is ambiguous (Torchy et al., 2015). xNuRD has an apparent molecular mass of 250–350 kDa, which is consistent with a previously described protein complex in *Xenopus laevis* oocytes containing HDACm and RBBP7/4 (Ryan et al., 1999) and a human NuRD complex (Zhang et al., 1998). However, it is smaller than a 1- to 1.5-MDa NuRD/Mi-2 complex purified from *Xenopus* egg extracts (Wade et al., 1998). Summing the masses of individual xNuRD subunits yields a value of about 500 kDa. The difference from the experimentally determined mass could be due to an elongated and non-spherical shape of xNuRD, contributing to a slower sedimentation during ultracentrifugation. Our data would therefore suggest that the active xNuRD complex probably contains only one copy of each subunit, with the possible exception of the small RBBP7/4 and MBD3 subunits.

Exclusive subunit utilization allows particular NuRD complexes to adopt particular biological functions. For instance, human MBD2 and MBD3 proteins are present in mutually exclusive NuRD complexes (Le Guezennec et al., 2006). MBD2-deficient mice are viable, whereas MBD3-deficient mice are early embryonic lethal (Hendrich et al., 2001), suggesting an essential and specific role for MBD3 during early mammalian development. Similarly, the presence of MTA1 in NuRD complexes correlates with metastatic growth of human tumor tissue and MTA3 with normal growth and differentiation (Ho and Crabtree, 2010). We have shown here that an MTA2-containing NuRD complex from *Xenopus laevis* eggs has an essential function in DNA replication in early *Xenopus* embryos, supporting a general role of MTA-containing NuRD complexes as regulators of cell growth and differentiation. Interestingly, MTA2 interacts with Tipin to recruit DNA polymerase α to chromatin and promote the stability of DNA replication forks in *Xenopus* egg extracts (Errico et al., 2014). However, this particular function of MTA2 does not involve the canonical NuRD complex, suggesting a separate role for MTA2 as part of the replication pausing complex (Errico et al., 2014).

We have observed that homologous NuRD complexes from human cells, in contrast to xNuRD, do not have DNA replication initiation activity. This functional difference could be due to differences in the subunit compositions between these NuRD complexes, including the specific incorporation of HDACm, CHD4, MTA2, and MBD3 in *Xenopus* and heterogeneous inclusion of HDAC1/2, CHD3/4, MTA1/2/3, and MBD2/3 and the presence of DOC1, in human cells.

### Functional Roles for Deacetylase Subunits

We have identified the maternal HDACm as the major deacetylase subunit of xNuRD. This 57-kDa protein is a class I histone lysine deacetylase enzyme, highly related to HDAC1 and characterized by a unique C-terminal domain (Ladomery et al., 1997, Ryan et al., 1999). HDACm accumulates during oogenesis and is maternally deposited in the egg (Ryan et al., 1999). After the MBT, expression of HDACm declines slowly, with mRNA becoming undetectable by the tailbud stage and protein by the swimming tadpole stage (Ladomery et al., 1997). We found that HDACm dissociates from the xNuRD complex after the MBT, concomitant with a decline of the associated DNA replication initiation activity. Thus, declining HDACm expression and complex dissociation may both underlie the loss of xNuRD DNA replication activity after the MBT, when initiation of DNA replication becomes dependent on the non-coding Y RNAs (Collart et al., 2011).

Following microinjection into oocytes, ectopic HDACm is transported into the nucleus (the germinal vesicle), causing a decondensation and transcriptional repression of lampbrush chromosomes (Smillie et al., 2004). Therefore, it is possible that the HDACm subunit of xNuRD also causes, or contributes to, the inhibition of zygotic transcription until the MBT.

Surprisingly, we found that treating fertilized eggs with the HDAC inhibitors SAHA or MS-275 did not result in significant inhibition of DNA synthesis before the MBT (data not shown), in contrast to the microinjection of xNuRD-specific antibodies. There may be technical reasons for this, such as poor compound dispersal or membrane permeability. However, it is also possible that the deacetylase activity of HDACm/xNuRD is not essential for DNA replication in the embryo before the MBT. In support of this idea, deacetylase inhibition by TSA treatment does not induce histone hyperacetylation and H1° gene expression in oocytes and early embryos before gastrulation (Almouzni et al., 1994), nor does it inhibit sperm chromatin replication in activated egg extracts (Lemaitre et al., 2005). In contrast, however, the HDAC activity of xNuRD is clearly required for the initiation of Y RNA-independent DNA replication in human somatic template nuclei *in vitro*. This might be explained by higher lysine acetylation levels in post-MBT embryos and somatic cells (Tsuchiya et al., 2014).

HDAC1 and HDAC2 activities also contribute to S phase progression in mammalian somatic cells by stabilizing DNA replication forks (Bhaskara, 2015, Bhaskara et al., 2013, Conti et al., 2010). Similar to HDAC1/2 inhibition, CHD4 depletion also results in delayed S phase progression in mammalian cells (Sims and Wade, 2011). Therefore, the functional roles for these somatic NuRD complexes during replication fork progression are separate from, but not inconsistent with, an essential role for xNuRD during replication initiation.

### DNA Replication Control during Early Development

The periods of xNuRD-dependent and Y RNA-dependent DNA replication during *Xenopus* development are distinct. We show here that inactivation of xNuRD results in an inhibition of DNA replication, a developmental delay, and embryo lethality before the MBT. In contrast, Y RNA inactivation has no effect before the MBT but results in the inhibition of DNA replication, developmental arrest, and embryo death after the MBT (Collart et al., 2011). Simultaneous inactivation of both xNuRD and Y RNA yields essentially the same pre-MBT phenotype as xNuRD inactivation alone but with a modest additive effect, suggesting a brief “handover period” of these two pathways during development, around the MBT.

Our discovery of an essential DNA replication function for the chromatin remodeling complex xNuRD provides a link between the control of DNA replication and the dynamics of the underlying chromatin structure. Replicating chromatin during the early cleavage stages is organized as aggregates of karyomeres, which are membrane-bound individual chromosomes that are more condensed than the canonical interphase nuclei appearing after MBT (Lemaitre et al., 1998, Montag et al., 1988). In these karyomeres, DNA replication actually overlaps with mitosis because individual chromosomes initiate DNA replication as separate units during telophase, allowing the very short duration of S phase before the MBT (Lemaitre et al., 1998). Therefore, xNuRD may play a crucial role in maintaining this condensed, fast-replicating, and transcriptionally silent chromatin environment during early cleavage stages. In the different chromatin environment of human late G1 phase nuclei, the remodeling activity of xNuRD could facilitate the initiation of new DNA replication forks in the absence of Y RNAs. It can thus be hypothesized that Y RNAs play a role in negotiating local and potentially repressive chromatin environments in human cell nuclei during the activation of chromosomal DNA replication origins. Future experiments will be required to test this idea.

We do not yet know whether the evolutionary conservation of the essential function of xNuRD extends beyond *Xenopus laevis*. However, based on the emergence of Y RNA-dependent pathways for DNA replication and embryo viability during early development in zebrafish and nematodes (Collart et al., 2011, Kowalski et al., 2015, Kowalski and Krude, 2015), it is likely that homologs of xNuRD may also exist in other vertebrate and non-vertebrate organisms.

The physiologically relevant targets of xNuRD activity are unknown. Future proteome-wide experiments should therefore be directed at identifying the acetyl-lysine residues on histone and non-histone proteins that are physiologically relevant for the HDACm activity of xNuRD. Similarly, genome-wide investigations may reveal relevant genomic locations for the nucleosome mobilization activity of the CHD4 subunit of xNuRD. The findings reported here, putting xNuRD at the crossroads of transcription repression and DNA replication, should expedite such investigations.

## Experimental Procedures

Further details and reagents used in this work can be found in the Supplemental Experimental Procedures.

### *Xenopus* Egg and Embryo Manipulations

Crude *Xenopus laevis* egg extracts were prepared after packing dejellied activated eggs at 900 × *g* for 1 min at 4°C by crushing through centrifugation at 20,000 × *g* for 15 min at 4°C (Blow and Laskey, 1986). After Y RNA degradation, extracts were fractionated over seven steps, and proteins were identified by mass spectrometry, as detailed in the Supplemental Experimental Procedures.

Embryos were obtained by artificial fertilization. They were maintained in 10% normal amphibian medium (NAM) (Slack, 1984) and staged as described previously (Nieuwkoop and Faber, 1975). *Xenopus* embryos were injected at the one-cell stage with 40 ng antisense morpholino oligonucleotides dissolved in water as described previously (control MO [coMO] or xY3MO; Collart et al., 2011) and/or with 65 ng protein of antiserum or 5 ng of purified antibodies. Embryos were imaged with a Leica M165FC dissecting scope with a DFC310FC digital camera using Leica LAS software v4.9.

Embryo extracts were prepared by triturating staged embryos in LB buffer (10 mM potassium 4-(2-hydroxyethyl)-1-piperazineethanesulfonic acid [K-HEPES] [pH 7.4], 150 mM NaCl, 2 mM EDTA, 0.5% Triton X-100, and Roche complete protease inhibitor cocktail) at 10 μL/embryo. Lipids and yolk were then removed from the lysate by extraction with an equal volume of Freon, and the aqueous phase was collected following centrifugation at 14,000 × *g* for 15 min at 4°C.

All Xenopus work complied fully with the UK Animals (Scientific Procedures) Act 1986 as implemented by the Francis Crick Institute.

### Human Cells and DNA Replication Initiation Assays

Human HeLaS3 and EJ30 cells were grown as proliferating monolayers as described previously (Krude, 2000, Krude et al., 1997). hESCs (WA09, WiCell) were cultured on Matrigel-coated Costar 100-mm plates (Corning Matrigel hESC-Qualified Matrix) in mTeSR1 medium (STEMCELL Technologies). Template nuclei were prepared from mimosine-arrested late G1 phase EJ30 cells as described previously (Krude, 2000).

DNA replication initiation reactions containing late G1 phase template nuclei, cytosolic extracts from proliferating HeLa cells, and a buffered ribo- and deoxyribonucleoside triphosphate and energy-regenerating mix were performed and analyzed by confocal immunofluorescence microscopy as detailed previously (Christov et al., 2006, Collart et al., 2011, Krude, 2000, Krude et al., 1997).

Endogenous Y RNAs were depleted from the extracts by antisense DNA oligonucleotides as detailed previously (Christov et al., 2006, Collart et al., 2011).

### Immunoprecipitation

For immunoprecipitations, *Xenopus laevis* egg or embryo extracts or human cell extracts were first precipitated with ammonium sulfate at 20%–45% saturation and fractionated by sucrose gradient ultracentrifugation to yield fractions 4–7 (for *Xenopus* extracts) or 6–8 (for human cell extracts) as detailed above. Immunoprecipitations and immunodepletions were performed with control rabbit IgG (sc-2027, Santa Cruz Biotechnology), rabbit anti-MTA2 (ab8106, Abcam), or anti-Vasa antibodies (sc30210, Santa Cruz), and protein A Dynabeads according to the supplier (Invitrogen). Precipitated beads were washed with PBS [pH 7.4] containing 0.02% Tween 20 prior to western blotting or mass spectrometry.

## Supplemental Information

Document S1. Supplemental Experimental Procedures and Figures S1–S6

Table S1. Isolation of the DNA Replication Initiation Activity from *Xenopus laevis* Egg Extract: Mass Spectrometry Data, Related to Figure 2 Mass spectrometry data from two repeats are presented on two separate sheets, together with the corresponding technical metadata. Quantitative Values (as normalized emPAI) and Exclusive Unique Peptide Counts are presented for analyses of the active fractions containing the peak of initiation activity and of a neighboring inactive fraction. Protein enrichment in the active fraction is also presented, determined by dividing quantitative values from the active by the inactive fractions. Data are ranked by enrichment, followed by quantitative values of the active fractions. Molecular weight data are derived from the Uniprot_080713 database and may differ from the experimentally determined values shown in the paper.

Table S2. Immunoprecipitation of MTA2-Associated Proteins from *Xenopus laevis* Egg Extract: Mass Spectrometry Data, Related to Figure 3 Mass spectrometry data are presented with the technical metadata. Quantitative Values (as normalized emPAI) and Exclusive Unique Peptide Counts are presented for analyses of the MTA2-IP and a control-IP using empty beads from *Xenopus laevis* fractionated egg extracts. Data are ranked by quantitative values of the MTA2-IP. Molecular weight data are derived from the Uniprot_2016 database and may differ from the experimentally determined values shown in the paper.

Table S3. Immunoprecipitation of Human NuRD Complexes: Mass Spectrometry Data, Related to Figure 4 Mass spectrometry data are presented with the technical metadata. Quantitative Values (as normalized emPAI) and Exclusive Unique Peptide Counts are presented for analyses of MTA2-IPs and a control-IPs using empty beads from partially fractionated human HeLa cell nuclear and cytosolic extracts. Data are ranked by quantitative values of the MTA2-IP of the nuclear extract. Molecular weight data are derived from the Uniprot_2016 database and may differ from the experimentally determined values shown in the main paper.

Table S4. Immunoprecipitation of MTA2-Associated Proteins from Staged *Xenopus laevis* Embryo Extracts: Mass Spectrometry Data, Related to Figure 6 Mass spectrometry data are presented with the technical metadata. Quantitative Values (as normalized emPAI) and Exclusive Unique Peptide Counts are presented for analyses of MTA2-IPs from pre-MBT stage 6 and post-MBT stage 18 *Xenopus laevis* partially fractionated embryo extracts. Data are ranked by quantitative values of the pre-MBT stage 6 MTA2-IP. Molecular weight data are derived from the Uniprot_2016 database and may differ from the experimentally determined values shown in the paper.

Movie S1. *Xenopus laevis* Development after Inhibition of xNuRD, Related to Figure 7 Phenotypes of developing *Xenopus laevis* embryos after microinjection of NuRD-specific antibodies. See Figure 6A for experimental details. From left to right, the four columns represent: (i) uninjected control embryos, embryos injected with (ii) HDACm antiserum, (iii) RBBP4/p48 antiserum, and (iv) a non-specific control antiserum. The three rows show experimental replicates of these injections from top to bottom. Embryos were injected into the animal pole at the 1 cell stage, and images taken every 3 min. The time-lapse movie was assembled from 300 frames covering a total of 15h of development, displayed at a rate of ten frames per second.

Movie S2. *Xenopus laevis* Development after Inhibition of xNuRD and Y3 RNA, Related to Figure 7 Phenotypes of developing *Xenopus laevis* embryos after microinjection of NuRD-specific antibodies and xY3 RNA-specific MOs. See Figure 6B for experimental details. From left to right, the four columns represent embryos injected with: (i) anti-GFP control antibodies and control MO, (ii) anti-GFP control antibodies and xY3 MO, (iii) anti-MBD3 antibodies and control MO, and (iv) anti-MBD3 and xY3 MO. The three rows show experimental replicates of these injections from top to bottom. Embryos were injected into the animal pole at the 1 cell stage, and images taken every 3 min. The time-lapse movie was assembled from 300 frames covering a total of 15h of development, displayed at a rate of ten frames per second.

Document S2. Article plus Supplemental Information
