# Supplementary material for: A NuRD Complex from Xenopus laevis Eggs Is Essential for DNA Replication during Early Embryogenesis
Source: Cell Rep. 2018 Feb 27;22(9):2265–78. doi: 10.1016/j.celrep.2018.02.015 (PMC5848848; doi:10.1016/j.celrep.2018.02.015)
Supplement: Document S1. Supplemental Experimental Procedures and Figures S1–S6 [file mmc1.pdf]

Cell Reports, Volume *22*

Supplemental Information

A NuRD Complex from *Xenopus laevis* Eggs Is Essential for DNA Replication during Early Embryogenesis

Christo P. Christov, Kevin S. Dingwell, Mark Skehel, Helen S. Wilkes, Julian E. Sale, James C. Smith, and Torsten Krude

Supplemental figures

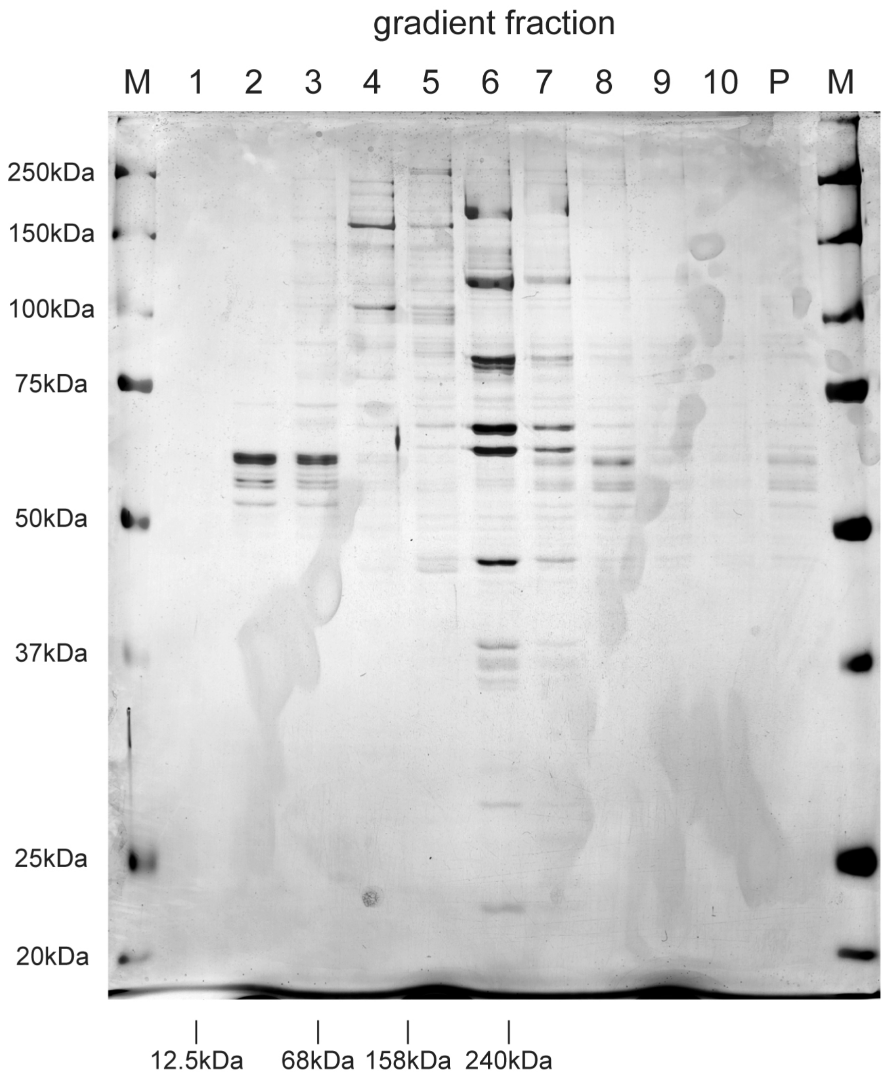

Figure S1. Polypeptide composition of the Y RNA-independent initiation factor from *Xenopus* egg extracts. Related to Figure 2.

Protein composition analysis of final sucrose minigradient fractions by SDS-PAGE and silver staining. Fraction numbers (1-10, collected from top to bottom of the gradient), pellet (P) and molecular weight markers (M) are indicated. Sedimentation positions of calibrator protein complexes (cytochrome C, 12.5kDa; bovine serum albumin, 68kDa; aldolase, 158kDa; catalase, 240kDa) are indicated at the bottom.

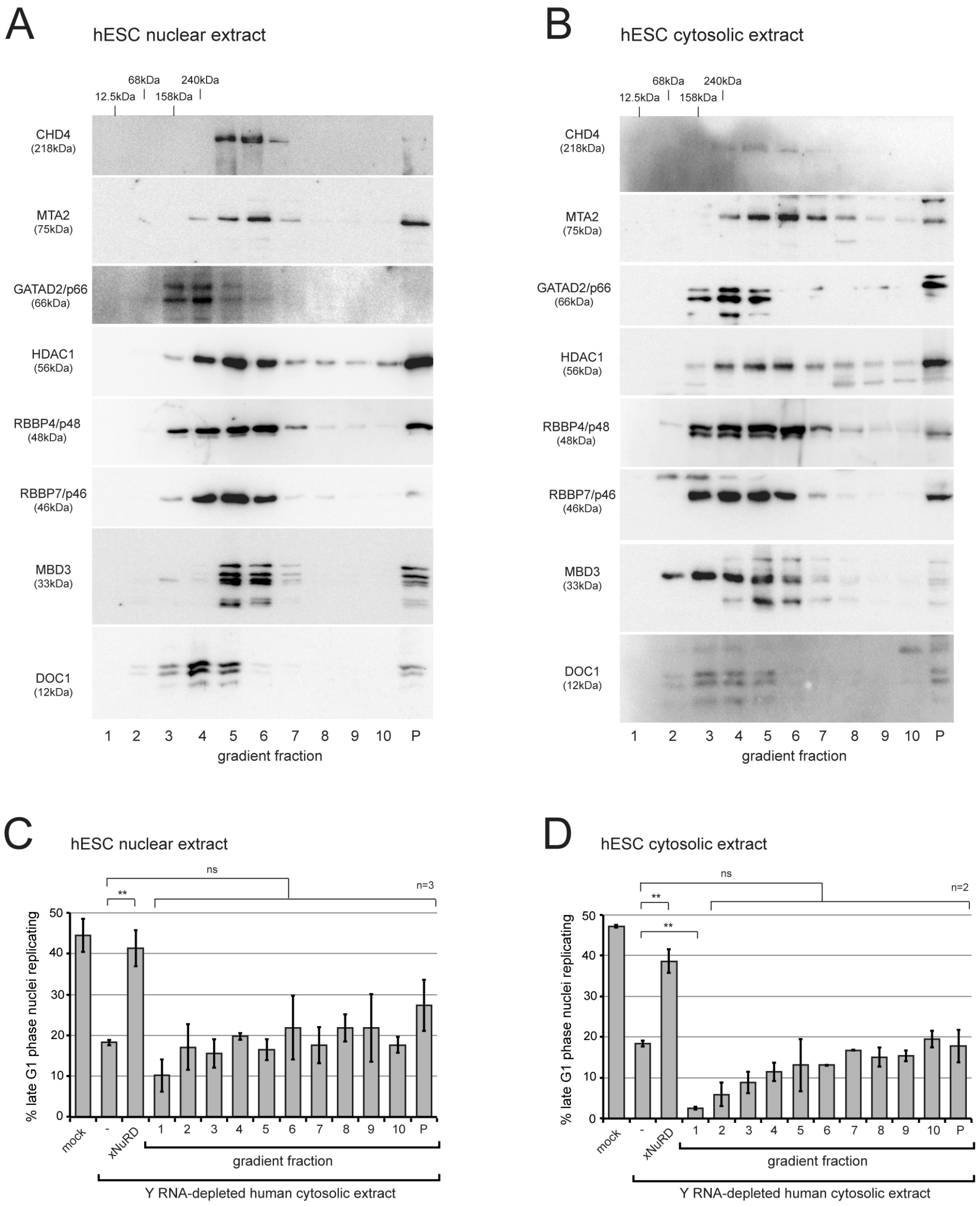

Figure S2. Structure and function of NuRD in human embryonic stem cell (hESC) extracts. Related to Figure 4.

Proliferating hESCs were fractionated into nuclear and cytosolic extracts, and each extract was partially sub-fractionated by precipitation with 20-45% ammonium sulphate and ultracentrifugation through preparative sucrose gradients. (A, B) Western blot analysis of indicated NuRD subunits in the preparative sucrose gradient fractions of the (A) nuclear and (B) cytosolic extracts. (C, D) Activity profiles of the sucrose fractions. Template nuclei were incubated in Y RNA-depleted human cytosolic extract supplemented with the indicated gradient fractions of the (C) nuclear and (D) cytosolic extracts. Mean values ± std of percentages of replicating template nuclei are plotted of n independent experiments. Brackets indicate results of T-tests (unpaired, two-tailed with unequal variance) of the positive control (xNuRD) and experimental samples against the Y RNA-depleted background (ns, not significant; ** $p \leq 0.02$).

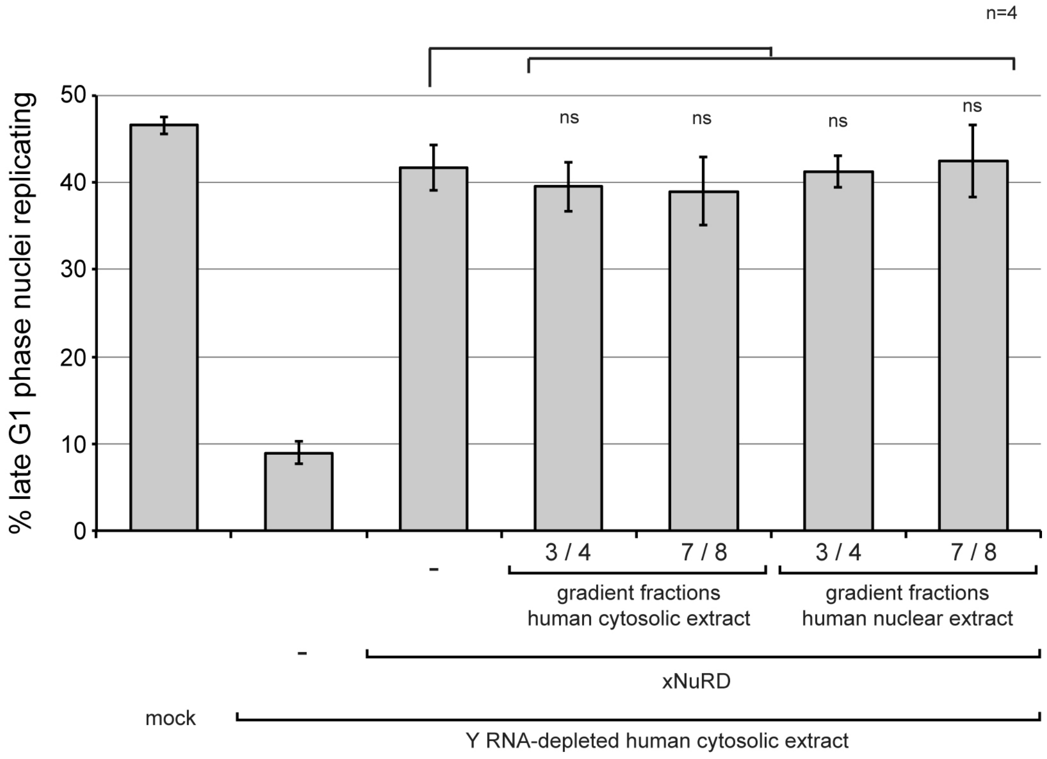

Figure S3. Human NuRD does not inhibit DNA replication. Related to Figure 4.

DNA replication initiation reactions. Template nuclei were incubated in Y RNA-depleted human cytosolic extract supplemented with or without xNuRD (15µg protein of the 20-45% the ammonium sulphate fraction; see Fig. 2) and the indicated sucrose gradient fractions of the cytosolic and nuclear HeLa cell extracts (8µl volumes, see Fig. 4). Mean values ± std of percentages of replicating template nuclei are plotted of n=4 independent experiments. Brackets indicate results of T-tests (unpaired, two-tailed with unequal variance) of the no-addition control against the experimental samples containing human NuRD fractions (ns, not significant).

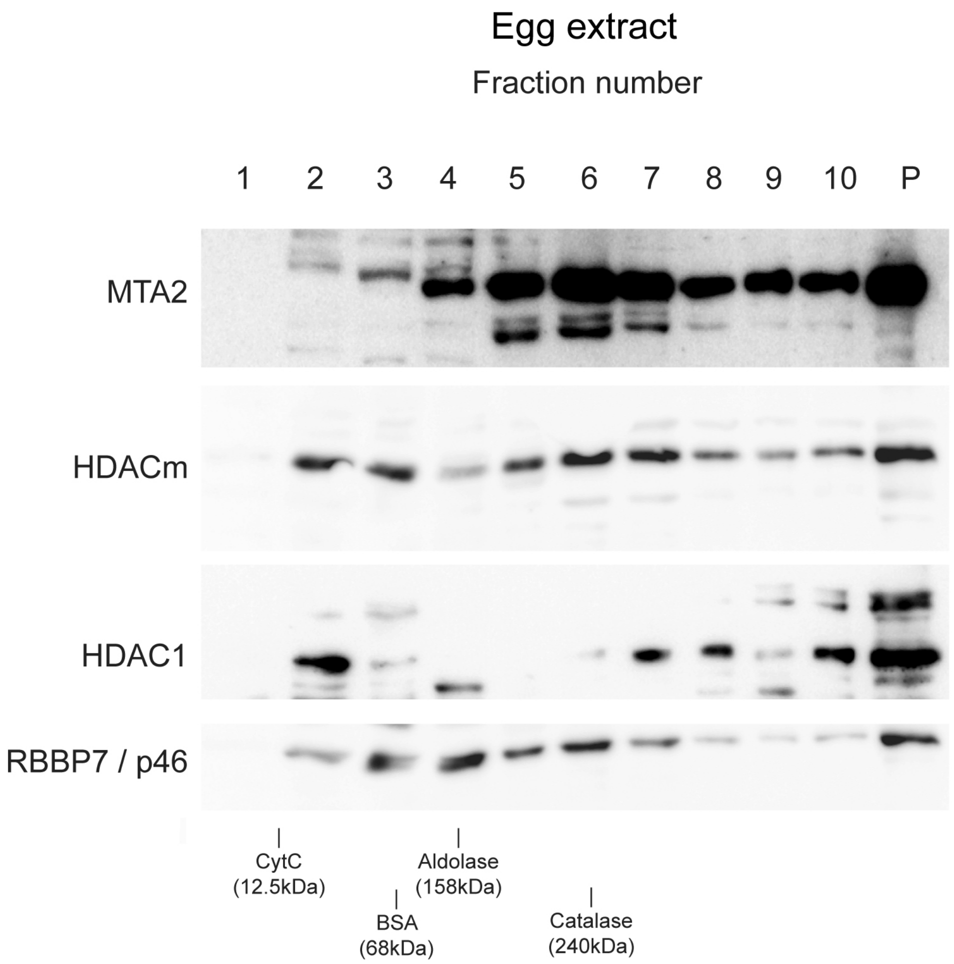

Figure S4. Complex formation of xNuRD in *Xenopus* egg extracts. Related to Figure 6.
xNuRD was partially purified by precipitation of egg extract with 20-45% ammonium sulphate and ultracentrifugation through preparative sucrose gradients. MTA2, HDACm, HDAC1 and RBBP7/p46 subunits were analysed by Western blot analysis of the preparative sucrose gradient fractions. Fraction numbers (1-10, collected from top to bottom of the gradient), the pellet fraction (P), and positions of sedimentation markers are indicated.

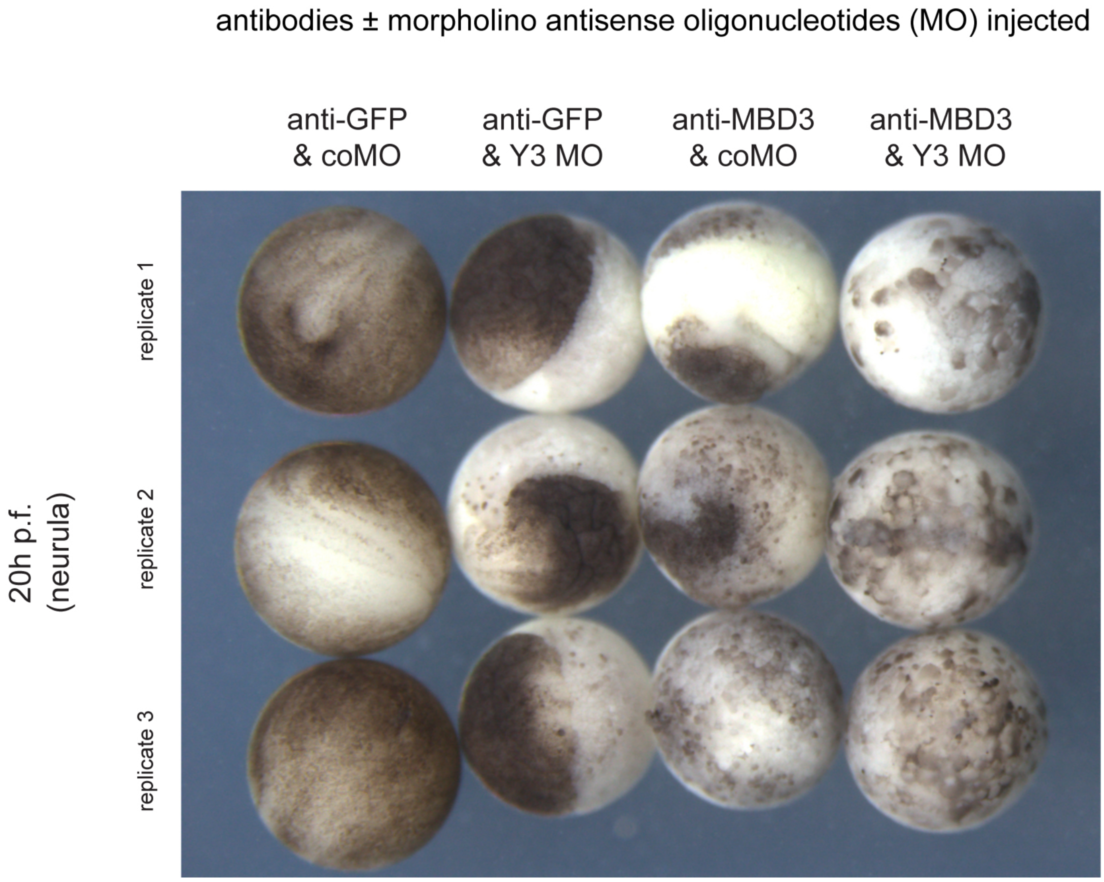

Figure S5. xNuRD is required for early development before the MBT and for embryo viability. Related to Figure 7.

Phenotypes of developing *Xenopus laevis* embryos after microinjection of NuRD-specific antibodies and Y3 RNA-specific antisense morpholino oligonucleotides (MOs). The indicated antibodies (5ng/embryo) and MOs (40 ng/embryo of coMO or xY3MO; Collart et al., 2011) were injected in the animal pole at the 1 cell stage, and representative embryos were photographed at 20 h post fertilisation (p.f.), when the control embryos had reached the neurula stage. Three experimental replicates of the injections are presented from top to bottom.

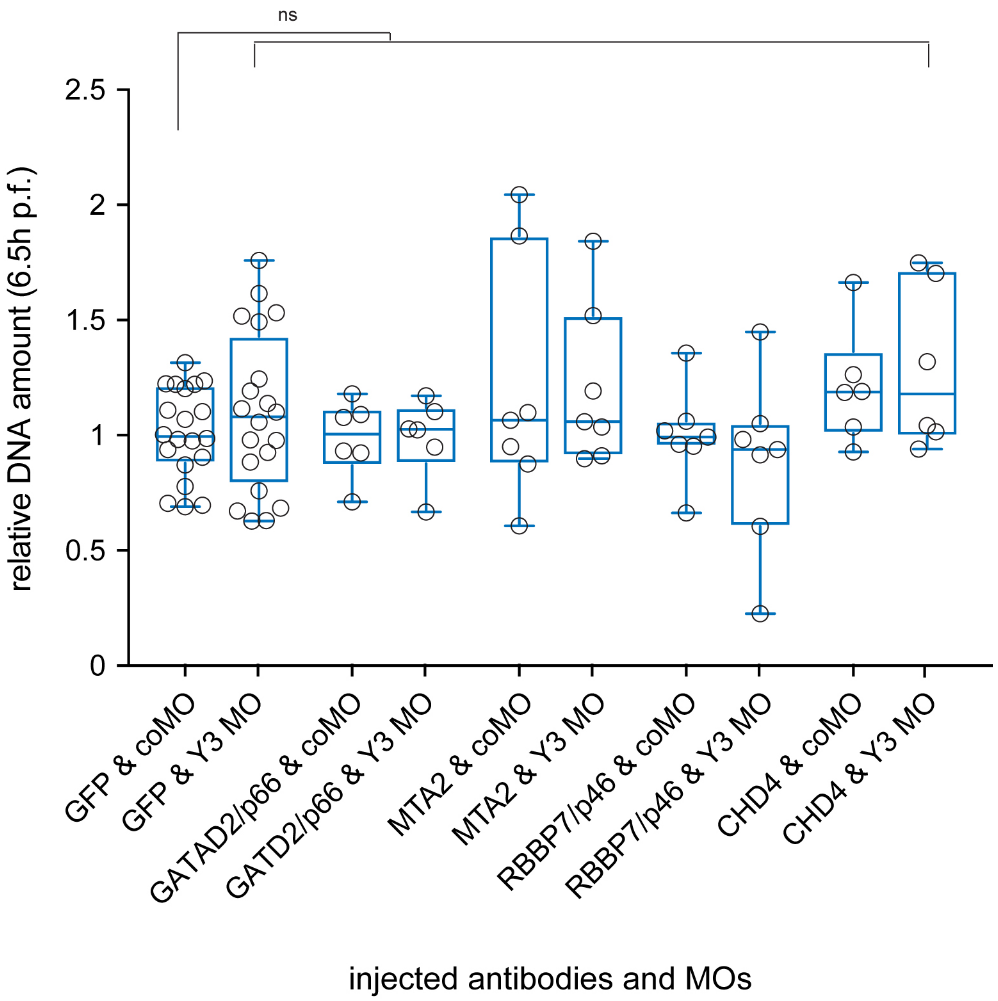

Figure S6. Embryonic DNA synthesis after microinjection with ineffective antibodies. Related to Figure 7.

Antibodies directed against control GFP and the indicated subunits of xNuRD (5ng/embryo) were (co)injected with the indicated MOs (40 ng/embryo of coMO or xY3MO; Collart et al., 2011) into the animal pole at the 1 cell stage. Total embryonic DNA of the experimental repeats was quantified relative to rRNA. Individual data sets were normalised to the mean of the control anti-GFP distributions, and plotted as box and whisker plots superimposed with individual data points. Results of T-tests (unpaired, two-tailed with unequal variance) of treated experimental samples against the controls are indicated (ns, not significant).

Supplemental Experimental Procedures

Antibodies and inhibitors

The following rabbit antibodies were used for both *Xenopus* and human cell extracts, after we had confirmed by Western blot analyses that they cross-react with polypeptides of the expected size in both species: anti-CHD4 (ab85620), anti-MTA1 (ab71153), anti-MTA2 (ab8106), anti-p66 alpha (ab87663), anti-HDAC1 (ab19845), anti-RbAp46 (ab3535), anti-RbAp48 (ab1765; all from Abcam). Rabbit anti-MBD3 (GTX116204, from GeneTex), anti-HDACm (C-pep, (Ryan et al., 1999)) and anti-nucleoplasmin (from Ron Laskey, University of Cambridge) were used exclusively for *Xenopus* extracts and embryo injections. Rabbit anti-CHD4 (ab72418), anti-MBD3 (ab157464), and anti-CDKA1/DOC1 (ab108290; all from Abcam) were used exclusively for Western blotting analyses of human cell extracts. Rabbit anti-GFP (ab6556, Abcam), mouse anti-actin (2A3, ab123034; Abcam), and control rabbit IgG (sc-2027; Santa Cruz) and rabbit anti-Vasa (d-260, sc30210; Santa Cruz) were used as negative controls for microinjection, replication assays and immunoprecipitations, respectively.

For Western blotting, we used nitrocellulose blotting membrane (GE Healthcare), secondary goat anti-rabbit HRP IgG (P0448, Dako), ECL Prime Western blotting detection reagent (RPN 2232, Amersham) and a Kodak Detection System for digital imaging.

The following HDAC inhibitors were dissolved in DMSO and used at the concentration specified: Trichostatin A, TSA (Cell Signaling Technology, 9950S), SAHA (Sigma, SML0061), and MS-275 (Sigma, EPS002).

Isolation of NuRD complexes: *Fractionation of Xenopus egg extracts*

Crude *Xenopus laevis* egg extracts were prepared after packing dejellied activated eggs at 900 x g for 1 min at 4°C by crushing through centrifugation at 20,000 x g for 15min at 4°C (Blow and Laskey, 1986). The entire soluble fraction between lipid plug and solid pellet was recovered and extracted twice with an equal volume of 1,1,2-Trichloro-1,2,2-trifluoroethane (Freon, Sigma-Aldrich) to remove lipids and yolk. At this stage, eggs extracts obtained from several females were pooled. Endogenous Y RNAs were degraded by addition of antisense DNA oligonucleotides to target endogenous RNAse H activity, as described previously (Collart et al., 2011).

Y RNA-depleted egg extracts were fractionated initially by ammonium sulphate precipitation. Initiation activity was present between 20% and 45% saturation. The 20-45% precipitate was dissolved and dialysed against replication buffer (20 mM K-HEPES pH 7.8, 100mM K acetate, 1mM DTT, 1mM EGTA), and subsequently loaded on 15% to 40% linear sucrose gradients prepared in replication buffer in 5ml tubes. Gradients were run in a Beckman Coulter OptimaMAX-XP ultracentrifuge for 18h at 124,000 x g at 4°C in a Beckman Coulter MLS-50 rotor. Gradients were manually fractionated into 10 soluble fractions (0.5ml volume). Pelleted material was resuspended in its residual volume and adjusted with replication buffer to 0.5ml. Initiation activity was present in fractions 4-7, which were then pooled and precipitated with ammonium sulphate at 100% saturation. The precipitate was dissolved in buffer A (50mM Tris pH 8.2, 150mM KCl, 1mM DTT, 1mM EGTA) and loaded on a Heparin Sepharose column, equilibrated in buffer A, using the Äkta pure FPLC platform (GE Healthcare). Protein fractions were step-eluted in buffer A adjusted to 500mM KCl (Hep500) and 1M KCl (Hep1M). Initiation activity was present in the Hep500 fraction. The Hep500 fraction was diluted to 150mM KCl and loaded onto a MonoQ column (5/50 GL, Pharmacia Biotech), equilibrated in buffer A. Proteins were eluted with a linear KCl gradient up to 600 mM KCl over a volume of 10ml. Fractions containing initiation activity were determined experimentally for each purification run (peaking typically between 340-380mM KCl), pooled and precipitated with ammonium sulphate at 100% saturation. The precipitate was dissolved and dialysed against replication buffer, and subsequently loaded on 15% to 40% linear sucrose minigradients prepared in replication buffer in 600μl tubes. Gradients were run for 18h at 124,000 x g at 4°C using tube adapters in an MLS-50 rotor, and fractionated into 10 fractions (60μl volumes), the residual pellet was resuspended in 60μl replication buffer. Activity peaked in fractions 6 and 7.

Isolation of human NuRD complexes: ***Fractionation of human cell extracts***

For isolation of hNuRD, HeLa cells and hESCs were fractionated into nuclei and cytosol by hypotonic washes, dounce homogenisation and centrifugation of the cell homogenate at 5,000 x g for 3mins as detailed previously (Krude et al., 1997).

The cytosolic supernatant was re-centrifuged at 16,000 x g for 15mins at 4°C. The supernatant served as the cytosolic extract and the pelleted debris was discarded.

The nuclei were extracted at 4°C in extraction buffer (20 mM K-HEPES pH 7.8, 600mM NaCl, 5mM K acetate, 0.5mM $MgCl_2$, 0.5mM DTT, 1mM EGTA) in a rotator for 40mins and centrifuged again at 16,000 x g for 15mins at 4°C. The supernatant provided the nuclear extract and the pelleted residual nuclear structures were discarded. Prior to use, nuclear extracts were dialysed against replication buffer.

Endogenous Y RNAs were depleted from the extracts by antisense DNA oligonucleotides as detailed previously (Christov et al., 2006; Collart et al., 2011). Depleted extracts were subsequently fractionated by ammonium sulphate precipitation at 20-45% saturation and sucrose gradient ultracentrifugation, exactly as detailed for *Xenopus* egg extracts above.

DNA Quantification in *Xenopus* embryos

DNA was isolated from *Xenopus laevis* embryos and quantified relative to rRNA as described (Rollins and Andrews, 1991), with modifications. In short, embryos were collected at stage 7 and lysed with SETS buffer (50mM Tris pH 7.5, 150mM NaCl, 10mM EDTA, 0.5% SDS; supplemented with 250µg/mL proteinase K) at 100µl/embryo. Lysates were incubated at 55˚C for 30 min and then extracted with an equal volume of phenol-chloroform-isoamyl alcohol, pH 7.9, and the aqueous phase collected following centrifugation using Phase Lock Gel heavy tubes (5Prime, Qanta Bio). DNA was precipitated with ethanol and resuspended in TE buffer (10mM Tris-Cl, pH 8.0, 1mM EDTA) at 20ul/embryo. 5 or 10µl volumes of DNA were run on 0.6% agarose gels in TAE buffer and the DNA-to-rRNA ratios were determined using Quantity One software (BioRad).

Mass spectrometry

Protein samples were prepared by two alternative methods: Polyacrylamide gel slices (1-2mm) containing the proteins were prepared for mass spectrometric analysis using the Janus liquid handling system (PerkinElmer, UK). Briefly, entire gel lanes were manually cut into 22 slices, and the excised protein gel pieces were placed in the wells of a 96-well microtitre plate and destained with 50% v/v acetonitrile and 50mM ammonium bicarbonate, reduced with 10mM DTT, and alkylated with 55mM iodoacetamide. After alkylation, proteins were digested with 6ng/µl trypsin (Promega, UK) overnight at 37°C. Alternatively, immunoprecipitated proteins bound directly to beads were reduced and alkylated as described above and then trypsinised, in 30µl of buffer containing 50mM ammonium

hydrogen carbonate pH 8.0 and 6ng/µl Trypsin). Digestion was performed for 60 min at 37°C in a thermomixer, shaking at 750 rpm. After the initial digestion, an additional 10µl of 6ng/µl Trypsin was added and the samples digested overnight at 37°C in a thermomixer, shaking at 750rpm.

The resulting peptides were extracted in 2% v/v formic acid, 2% v/v acetonitrile. Digests were analysed by nano-scale capillary LC-MS/MS using an Ultimate U3000 HPLC (ThermoScientific Dionex, San Jose, USA) to deliver a flow of approximately 300nl/min. A C18 Acclaim PepMap100 5µm, 100µm x 20mm nanoViper (ThermoScientific Dionex, San Jose, USA) trapped the peptides prior to separation on a C18 Acclaim PepMap100 3µm, 75µm x 250mm nanoViper (ThermoScientific Dionex, San Jose, USA). Peptides were eluted with a 60min gradient of acetonitrile (2% to 80%). The analytical column outlet was directly interfaced via a nano-flow electrospray ionisation source, with a hybrid quadrupole orbitrap mass spectrometer (Q-Exactive Plus Orbitrap, ThermoScientific, San Jose, USA). Data dependent analysis was carried out, using a resolution of 30,000 for the full MS spectrum, followed by ten MS/MS spectra. MS spectra were collected over a m/z range of 300–2000. MS/MS scans were collected using a threshold energy of 27 for higher energy collisional dissociation (HCD). LC-MS/MS data were then searched against a protein database (UniProt KB) using the Mascot search engine programme (Matrix Science, UK) (Perkins et al., 1999). Database search parameters were set with a precursor tolerance of 10ppm and a fragment ion mass tolerance of 0.8Da. One missed enzyme cleavage was allowed and variable modifications for oxidized methionine, carbamidomethyl cysteine, pyroglutamic acid, phosphorylated serine, threonine and tyrosine, and methyl arginine were included. MS/MS data were validated using the Scaffold programme (Proteome Software Inc., USA) (Keller et al., 2002). All data were additionally interrogated manually.

Supplemental References

Blow, J.J., and Laskey, R.A. (1986). Initiation of DNA replication in nuclei and purified DNA by a cell-free extract of Xenopus eggs. Cell *47*, 577-587.

Christov, C.P., Gardiner, T.J., Szüts, D., and Krude, T. (2006). Functional requirement of noncoding Y RNAs for human chromosomal DNA replication. Mol Cell Biol *26*, 6993-7004.

Collart, C., Christov, C.P., Smith, J.C., and Krude, T. (2011). The midblastula transition defines the onset of Y RNA-dependent DNA replication in Xenopus laevis. Mol Cell Biol *31*, 3857-3870.

Keller, A., Nesvizhskii, A.I., Kolker, E., and Aebersold, R. (2002). Empirical statistical model to estimate the accuracy of peptide identifications made by MS/MS and database search. Anal Chem *74*, 5383-5392.

Krude, T., Jackman, M., Pines, J., and Laskey, R.A. (1997). Cyclin/Cdk-dependent initiation of DNA replication in a human cell-free system. Cell *88*, 109-119.

Perkins, D.N., Pappin, D.J., Creasy, D.M., and Cottrell, J.S. (1999). Probability-based protein identification by searching sequence databases using mass spectrometry data. Electrophoresis *20*, 3551-3567.

Rollins, M.B., and Andrews, M.T. (1991). Morphogenesis and regulated gene activity are independent of DNA replication in Xenopus embryos. Development *112*, 559-569.

Ryan, J., Llinas, A.J., White, D.A., Turner, B.M., and Sommerville, J. (1999). Maternal histone deacetylase is accumulated in the nuclei of Xenopus oocytes as protein complexes with potential enzyme activity. J Cell Sci *112*, 2441-2452.
